# (*Z*)-1-Chloro-1-[2-(2-nitro­phen­yl)hydrazinyl­idene]propan-2-one

**DOI:** 10.1107/S1600536812049938

**Published:** 2012-12-12

**Authors:** Rami Y. Morjan, Bassam A. Abu Thaher, Dieter Schollmeyer, Adel M. Awadallah, John M. Gardiner

**Affiliations:** a Chemistry Department, Faculty of Science, Islamic University of Gaza, PO Box 108, Gaza, Palestine; b Department of Organic Chemistry, Johannes Gutenberg-University Mainz, Duesbergweg 10-14, D-55099 Mainz, Germany; c Manchester Institute of Biotechnology, School of Chemistry and EPS, The University of Manchester, Manchester M1 7DN, England

## Abstract

The title mol­ecule, C_9_H_8_ClN_3_O_3_, lies on a mirror plane. Intra­molecular N—H⋯O and N—H⋯Cl hydrogen bonds occur. One of the nitro O atoms is disordered (site occupancy ratio = 0.40:0.10).

## Related literature

For details of the synthesis and for the importance of hydrazonoyl halides in organic synthesis and their biological activity and metabolism, see: Awadallah *et al.* (2006 ▶, 2008 ▶); Budarina *et al.* (2007 ▶); Shawalia *et al.* (2009 ▶); Thaher *et al.* (2002 ▶).
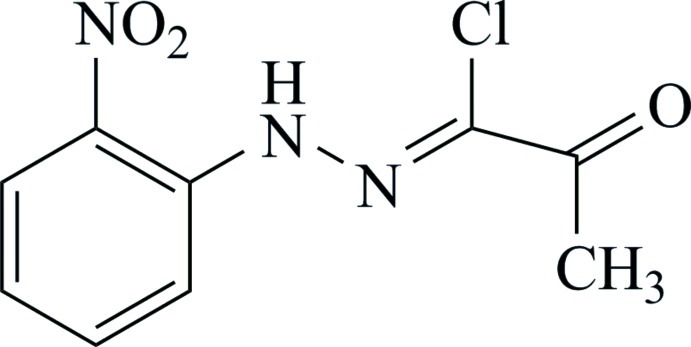


## Experimental

### 

#### Crystal data

C_9_H_8_ClN_3_O_3_
*M* *_r_* = 241.63
Orthorhombic, 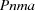

*a* = 14.1344 (10) Å
*b* = 6.5420 (5) Å
*c* = 11.3748 (8) Å
*V* = 1051.80 (13) Å^3^
*Z* = 4
Mo *K*α radiation
μ = 0.36 mm^−1^
*T* = 173 K
0.25 × 0.13 × 0.05 mm

#### Data collection

Bruker APEXII diffractometer
6908 measured reflections
1365 independent reflections
1003 reflections with *I* > 2σ(*I*)
*R*_int_ = 0.045

#### Refinement

*R*[*F* ^2^ > 2σ(*F* ^2^)] = 0.044
*wR*(*F* ^2^) = 0.121
*S* = 1.05
1365 reflections
109 parameters
H-atom parameters constrained
Δρ_max_ = 0.58 e Å^−3^
Δρ_min_ = −0.28 e Å^−3^

### 

Data collection: *APEX2* (Bruker, 2006 ▶); cell refinement: *SAINT* (Bruker, 2006 ▶); data reduction: *SAINT*; program(s) used to solve structure: *SIR97* (Altomare *et al.*, 1999 ▶); program(s) used to refine structure: *SHELXL97* (Sheldrick, 2008 ▶); molecular graphics: *PLATON* (Spek, 2009 ▶); software used to prepare material for publication: *PLATON*.

## Supplementary Material

Crystal structure: contains datablock(s) I, global. DOI: 10.1107/S1600536812049938/rn2108sup1.cif

Structure factors: contains datablock(s) I. DOI: 10.1107/S1600536812049938/rn2108Isup2.hkl

Supplementary material file. DOI: 10.1107/S1600536812049938/rn2108Isup3.cml

Additional supplementary materials:  crystallographic information; 3D view; checkCIF report

## Figures and Tables

**Table 1:** Hydrogen-bond geometry (Å, °)

*D*—H⋯*A*	*D*—H	H⋯*A*	*D*⋯*A*	*D*—H⋯*A*
N10—H10⋯Cl1	0.93	2.44	2.912 (2)	111
N10—H10⋯O8	0.93	2.00	2.616 (3)	122

**Fig. 1.**
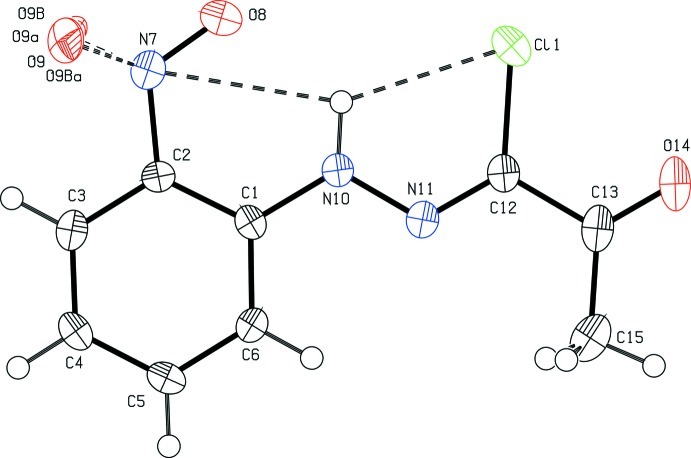
View of the title compound. Displacement ellipsoids are drawn at the 50% probability level. H atoms are depicted as circles of arbitrary size. Hydrogen bonds and bonds to disordered oxygen atom O9 are shown with dashed lines.

## supplementary crystallographic information

### Comment

Hydrozonyl halides are considered as an important precursor in organic synthesis. They have been used extensively as starting material for *in situ* generation of 1,3-dipoles which in turn can be reacted with a wide range of organic species to generate five and six membered heterocyclic ring compounds. The molecule has two intermolecular NH···O and NH···Cl hydrogen bonds (Table 1) and distance between the molecules is 3.2710 (1) Å. The ring centers are ~2.87 Å apart. The packing is characterized by parallel molecules (perpendicular distance between the centre of gravity of the aromatic rings 3.2710 (1) Å). The molecule is planar and in order to calculate the distance a plane is required to be defined through a set of atoms (phenyl rings) and the distance of any atom of the nearest symmetry related molecules to this plane is then calculated. The distance between the symmetrical related phenyl rings is 3.2710 (1) Å but due to the slippage of centres of gravity of the rings (2.866 Å) there is no π-π interaction between the two rings.

### Experimental

2-Nitroaniline (0.1 mol) was dissolved in cold aqueous hydrochloric acid (80 ml, 5 N). To this solution was added dropwise a solution of sodium nitrite (7.6 g, 0.1 mol) in water (25 ml) with efficient stirring at 273–278 K. Stirring was continued for 20–30 min. The resulting freshly prepared solution of 2-nitrobenzendiazonium chloride was poured into a vigorously stirred cold solution (268 K) of 3-chloroacetylacetone (13.5 g, 0.1 mol) in pyridine/ water (160 ml 1:1 *v*/*v*). Stirring was continued until a solid precipitate was formed (10–20 min.). The reaction mixture was then diluted with cold water (200 ml), the solid product formed was collected, washed several times with cold water, dried and washed with ethanol. A small amount of the product was dissolved in DMF and left at room temperature for 2 days. Yellow needle crystals were isolated, m.p. 393 K.

### Refinement

Hydrogen atoms attached to carbons were placed at calculated positions with C—H = 0.95 Å (aromatic) or 0.98–0.99 Å (*sp*^3^ C-atom). Hydrogen atoms attached to nitrogen were located in diff. Fourier maps. All H atoms were refined in the riding-model approximation with isotropic displacement parameters (set at 1.2–1.5 times of the *U*_eq_ of the parent atom). The NO_2_ group is disorderd where one oxygen atom just off the mirror plane has two positions with relative site occupancies set to 0.4, (O9) and 0.1 (O9B) which generate a further two positions (their mirror images) with the same occupancies.

### Crystal data

**Table:**

C_9_H_8_ClN_3_O_3_	*F*(000) = 496
*M**_r_* = 241.63	*D*_x_ = 1.526 Mg m^−^^3^
Orthorhombic, *P**n**m**a*	Mo *K*α radiation, λ = 0.71073 Å
Hall symbol: -P 2ac 2n	Cell parameters from 1147 reflections
*a* = 14.1344 (10) Å	θ = 2.3–25.8°
*b* = 6.5420 (5) Å	µ = 0.36 mm^−^^1^
*c* = 11.3748 (8) Å	*T* = 173 K
*V* = 1051.80 (13) Å^3^	Needle, yellow
*Z* = 4	0.25 × 0.13 × 0.05 mm

### Data collection

**Table:**

Bruker APEXII diffractometer	1003 reflections with *I* > 2σ(*I*)
Radiation source: sealed Tube	*R*_int_ = 0.045
Graphite monochromator	θ_max_ = 27.9°, θ_min_ = 2.3°
CCD scan	*h* = −18→18
6908 measured reflections	*k* = −8→8
1365 independent reflections	*l* = −14→14

### Refinement

**Table:**

Refinement on *F*^2^	Primary atom site location: structure-invariant direct methods
Least-squares matrix: full	Secondary atom site location: difference Fourier map
*R*[*F*^2^ > 2σ(*F*^2^)] = 0.044	Hydrogen site location: inferred from neighbouring sites
*wR*(*F*^2^) = 0.121	H-atom parameters constrained
*S* = 1.05	*w* = 1/[σ^2^(*F*_o_^2^) + (0.0651*P*)^2^ + 0.198*P*] where *P* = (*F*_o_^2^ + 2*F*_c_^2^)/3
1365 reflections	(Δ/σ)_max_ = 0.001
109 parameters	Δρ_max_ = 0.58 e Å^−^^3^
0 restraints	Δρ_min_ = −0.28 e Å^−^^3^

### Special details

**Table:**

Geometry. All e.s.d.'s (except the e.s.d. in the dihedral angle between two l.s. planes) are estimated using the full covariance matrix. The cell e.s.d.'s are taken into account individually in the estimation of e.s.d.'s in distances, angles and torsion angles; correlations between e.s.d.'s in cell parameters are only used when they are defined by crystal symmetry. An approximate (isotropic) treatment of cell e.s.d.'s is used for estimating e.s.d.'s involving l.s. planes.
Refinement. Refinement of *F*^2^ against ALL reflections. The weighted *R*-factor *wR* and goodness of fit *S* are based on *F*^2^, conventional *R*-factors *R* are based on *F*, with *F* set to zero for negative *F*^2^. The threshold expression of *F*^2^ > σ(*F*^2^) is used only for calculating *R*-factors(gt) *etc*. and is not relevant to the choice of reflections for refinement. *R*-factors based on *F*^2^ are statistically about twice as large as those based on *F*, and *R*- factors based on ALL data will be even larger.

### Fractional atomic coordinates and isotropic or equivalent isotropic displacement parameters (Å^2^)

**Table:**

	*x*	*y*	*z*	*U*_iso_*/*U*_eq_	Occ. (<1)
Cl1	0.17306 (5)	0.2500	0.30944 (6)	0.0416 (3)	
C1	−0.00994 (17)	0.2500	0.0079 (2)	0.0227 (5)	
C2	0.02943 (17)	0.2500	−0.1054 (2)	0.0240 (5)	
C3	−0.02668 (19)	0.2500	−0.2057 (2)	0.0276 (6)	
H3	0.0012	0.2500	−0.2798	0.033*	
C4	−0.12361 (18)	0.2500	−0.1949 (2)	0.0294 (6)	
H4	−0.1617	0.2500	−0.2616	0.035*	
C5	−0.16419 (17)	0.2500	−0.0832 (2)	0.0302 (6)	
H5	−0.2297	0.2500	−0.0759	0.036*	
C6	−0.10892 (18)	0.2500	0.0161 (2)	0.0268 (6)	
H6	−0.1375	0.2500	0.0898	0.032*	
N7	0.13162 (16)	0.2500	−0.1243 (2)	0.0357 (6)	
O8	0.18551 (13)	0.2500	−0.04010 (17)	0.0413 (5)	
O9	0.1605 (11)	0.2922 (10)	−0.2234 (15)	0.031 (9)	0.40
O9B	0.156 (5)	0.135 (13)	−0.225 (6)	0.033 (15)	0.10
N10	0.04475 (14)	0.2500	0.10920 (17)	0.0270 (5)	
H10	0.1106	0.2500	0.1091	0.040*	
N11	0.00142 (15)	0.2500	0.21389 (18)	0.0259 (5)	
C12	0.05021 (18)	0.2500	0.3081 (2)	0.0277 (6)	
C13	0.0018 (2)	0.2500	0.4244 (2)	0.0362 (7)	
O14	0.04865 (17)	0.2500	0.51438 (17)	0.0502 (6)	
C15	−0.1042 (2)	0.2500	0.4226 (3)	0.0517 (9)	
H15A	−0.1274	0.2500	0.5019	0.078*	
H15B	−0.1265	0.1302	0.3826	0.078*	

### Atomic displacement parameters (Å^2^)

**Table:**

	*U*^11^	*U*^22^	*U*^33^	*U*^12^	*U*^13^	*U*^23^
Cl1	0.0303 (4)	0.0640 (5)	0.0305 (4)	0.000	−0.0087 (3)	0.000
C1	0.0224 (12)	0.0260 (13)	0.0198 (11)	0.000	−0.0032 (9)	0.000
C2	0.0186 (11)	0.0320 (14)	0.0215 (12)	0.000	0.0008 (9)	0.000
C3	0.0321 (13)	0.0315 (14)	0.0192 (12)	0.000	0.0016 (10)	0.000
C4	0.0270 (13)	0.0376 (15)	0.0236 (13)	0.000	−0.0081 (11)	0.000
C5	0.0196 (11)	0.0413 (16)	0.0297 (14)	0.000	−0.0047 (10)	0.000
C6	0.0234 (12)	0.0373 (15)	0.0197 (12)	0.000	0.0029 (10)	0.000
N7	0.0293 (12)	0.0544 (16)	0.0235 (12)	0.000	0.0018 (10)	0.000
O8	0.0236 (10)	0.0659 (15)	0.0344 (11)	0.000	−0.0027 (9)	0.000
O9	0.031 (2)	0.03 (3)	0.028 (2)	0.002 (5)	0.0129 (16)	0.006 (6)
O9B	0.040 (10)	0.03 (4)	0.027 (8)	−0.010 (18)	0.001 (7)	−0.016 (19)
N10	0.0214 (10)	0.0401 (13)	0.0194 (10)	0.000	−0.0004 (8)	0.000
N11	0.0265 (11)	0.0307 (12)	0.0206 (10)	0.000	0.0007 (9)	0.000
C12	0.0277 (13)	0.0352 (15)	0.0203 (13)	0.000	−0.0011 (10)	0.000
C13	0.0430 (16)	0.0457 (18)	0.0199 (12)	0.000	0.0012 (12)	0.000
O14	0.0572 (14)	0.0749 (17)	0.0186 (10)	0.000	−0.0033 (10)	0.000
C15	0.0446 (18)	0.079 (3)	0.0315 (17)	0.000	0.0111 (15)	0.000

### Geometric parameters (Å, º)

**Table:**

Cl1—C12	1.736 (3)	N7—O9	1.230 (16)
C1—N10	1.388 (3)	N7—O9^i^	1.230 (16)
C1—C6	1.402 (3)	N7—O9B	1.42 (6)
C1—C2	1.403 (3)	N7—O9B^i^	1.42 (6)
C2—C3	1.390 (3)	O9—O9^i^	0.553 (13)
C2—N7	1.460 (3)	O9B—O9B^i^	1.50 (17)
C3—C4	1.376 (4)	N10—N11	1.339 (3)
C3—H3	0.9300	N10—H10	0.9315
C4—C5	1.394 (4)	N11—C12	1.275 (3)
C4—H4	0.9300	C12—C13	1.489 (4)
C5—C6	1.374 (3)	C13—O14	1.220 (3)
C5—H5	0.9300	C13—C15	1.497 (4)
C6—H6	0.9300	C15—H15A	0.9600
N7—O8	1.224 (3)	C15—H15B	0.9600
			
N10—C1—C6	120.0 (2)	O8—N7—O9B^i^	119 (3)
N10—C1—C2	122.8 (2)	O9—N7—O9B^i^	19 (3)
C6—C1—C2	117.2 (2)	O9^i^—N7—O9B^i^	45 (4)
C3—C2—C1	121.8 (2)	O9B—N7—O9B^i^	64 (7)
C3—C2—N7	116.3 (2)	O8—N7—C2	120.0 (2)
C1—C2—N7	121.8 (2)	O9—N7—C2	117.6 (8)
C4—C3—C2	119.7 (2)	O9^i^—N7—C2	117.6 (8)
C4—C3—H3	120.2	O9B—N7—C2	111 (2)
C2—C3—H3	120.2	O9B^i^—N7—C2	111 (2)
C3—C4—C5	119.4 (2)	O9^i^—O9—N7	77.0 (3)
C3—C4—H4	120.3	N7—O9B—O9B^i^	58 (4)
C5—C4—H4	120.3	N11—N10—C1	118.9 (2)
C6—C5—C4	121.1 (2)	N11—N10—H10	117.3
C6—C5—H5	119.5	C1—N10—H10	123.8
C4—C5—H5	119.5	C12—N11—N10	120.0 (2)
C5—C6—C1	120.8 (2)	N11—C12—C13	119.9 (2)
C5—C6—H6	119.6	N11—C12—Cl1	123.26 (19)
C1—C6—H6	119.6	C13—C12—Cl1	116.89 (18)
O8—N7—O9	120.7 (8)	O14—C13—C12	119.7 (3)
O8—N7—O9^i^	120.7 (8)	O14—C13—C15	123.7 (3)
O9—N7—O9^i^	26.0 (6)	C12—C13—C15	116.6 (2)
O8—N7—O9B	119 (3)	C13—C15—H15A	109.2
O9—N7—O9B	45 (4)	C13—C15—H15B	109.6
O9^i^—N7—O9B	19 (3)	H15A—C15—H15B	109.5
			
N10—C1—C2—C3	180.0	C3—C2—N7—O9B^i^	35 (4)
C6—C1—C2—C3	−0.0	C1—C2—N7—O9B^i^	−145 (4)
N10—C1—C2—N7	−0.0	O8—N7—O9—O9^i^	97.9 (3)
C6—C1—C2—N7	180.0	O9B—N7—O9—O9^i^	−4 (5)
C1—C2—C3—C4	0.0	O9B^i^—N7—O9—O9^i^	−172 (11)
N7—C2—C3—C4	180.0	C2—N7—O9—O9^i^	−96.9 (3)
C2—C3—C4—C5	−0.0	O8—N7—O9B—O9B^i^	−110 (2)
C3—C4—C5—C6	0.0	O9—N7—O9B—O9B^i^	−4 (6)
C4—C5—C6—C1	−0.0	O9^i^—N7—O9B—O9B^i^	−9 (13)
N10—C1—C6—C5	180.0	C2—N7—O9B—O9B^i^	104 (3)
C2—C1—C6—C5	0.0	C6—C1—N10—N11	−0.0
C3—C2—N7—O8	180.0	C2—C1—N10—N11	180.0
C1—C2—N7—O8	0.0	C1—N10—N11—C12	180.0
C3—C2—N7—O9	14.7 (4)	N10—N11—C12—C13	180.0
C1—C2—N7—O9	−165.3 (4)	N10—N11—C12—Cl1	−0.0
C3—C2—N7—O9^i^	−14.7 (4)	N11—C12—C13—O14	180.0
C1—C2—N7—O9^i^	165.3 (4)	Cl1—C12—C13—O14	0.0
C3—C2—N7—O9B	−35 (4)	N11—C12—C13—C15	−0.0
C1—C2—N7—O9B	145 (4)	Cl1—C12—C13—C15	180.0

Symmetry code: (i) *x*, −*y*+1/2, *z*.

### Hydrogen-bond geometry (Å, º)

**Table:**

*D*—H···*A*	*D*—H	H···*A*	*D*···*A*	*D*—H···*A*
N10—H10···Cl1	0.93	2.44	2.912 (2)	111
N10—H10···O8	0.93	2.00	2.616 (3)	122
